# Improved Hemocompatibility on Superhemophobic Micro–Nano-Structured Titanium Surfaces

**DOI:** 10.3390/bioengineering10010043

**Published:** 2022-12-29

**Authors:** Vignesh K. Manivasagam, Ketul C. Popat

**Affiliations:** 1 Department of Mechanical Engineering, Colorado State University, Fort Collins, CO 80523, USA; 2 School of Biomedical Engineering, Colorado State University, Fort Collins, CO 80523, USA; 3 School of Advanced Materials Discovery, Colorado State University, Fort Collins, CO 80523, USA

**Keywords:** titanium implant surface, micro–nano surface topography, superhydrophobic, superhemophobic, hemocompatible

## Abstract

Blood-contacting titanium-based implants such as endovascular stents and heart valve casings are prone to blood clotting due to improper interactions at the surface level. In complement, the current clinical demand for cardiovascular implants is at a new apex. Hence, there is a crucial necessity to fabricate an implant with optimal mechanical properties and improved blood compatibility, while simultaneously interacting differentially with cells and other microbial agents. The present study intends to develop a superhydrophobic implant surface with the novel micro–nano topography, developed using a facile thermochemical process. The surface topography, apparent contact angle, and crystal structure are characterized on different surfaces. The hemo/blood compatibility on different surfaces is assessed by evaluating hemolysis, fibrinogen adsorption, cell adhesion and identification, thrombin generation, complement activation, and whole blood clotting kinetics. The results indicate that the super-hemo/hydrophobic micro–nano titanium surface improved hemocompatibility by significantly reducing fibrinogen adsorption, platelet adhesion, and leukocyte adhesion. Thus, the developed surface has high potential to be used as an implant. Further studies are directed towards analyzing the mechanisms causing the improved hemocompatibility of micro/nano surface features under dynamic in vitro and in vivo conditions.

## 1. Introduction

In recent times, a considerable increase in the demand for cardiovascular implants to repair and replace damaged tissues has been noted [1,2]. Titanium and titanium alloys are preferred as an implant material owing to its suitable mechanical properties, corrosion resistance, and bio/hemo-compatibility [3,4]. Titanium and titanium alloys have been preferred for several cardiovascular implants such as endovascular stents, neurovascular flow diverters, structural heart devices, and heart valve casing [5,6,7]. However, these implants may fail due to inappropriate interactions at the surface level with blood and its components, leading to the initiation of the coagulation cascade followed by thrombus formation and inflammation [8,9]. Clinically, patients with cardiovascular implants are prescribed the use of systemic anticoagulant drugs and anti-platelets to avoid thrombus formation on the implant surface [10,11]. However, studies have shown that the overuse of these drugs can lead to internal bleeding complications, notably in patients who are already prone to high bleeding risks and in elderly patients [11,12]. In addition, anticoagulant therapy has also shown variable responses due to poor adherence and genetic variability [13,14]. Hence, there is a critical need to develop cardiovascular implants with suitable surface properties that prevent thrombus formation. 

Implant surfaces are prone to thrombus formation due to the hemodynamic alteration of blood flow and improper interactions by the implant compared to the native endothelial layer-covered arteries [15]. Once the blood flows over the implant surface, there is a rapid increase in plasma protein (fibrinogen and von Willebrand factor) adsorption on the surface [16,17]. These proteins alleviate the coagulation cascade and thrombin formation. Fibrinogen adsorption further promotes the adhesion and activation of platelets. Simultaneously, there is contact activation of the intrinsic coagulation pathway by adsorbing factor XII and its subsequent change in confrontation and activation. Activated FXIIa initiates the activation of other factors, leading to the formation of thrombin and complement activation on the surface. Thrombin reacts with fibrinogen and forms a mesh to trap activated platelets and red blood cells (RBCs) to form blood clot. Additionally, the activation of the coagulation cascade is greatly interconnected with platelet and leukocyte (WBC) adhesion and activation [4,18,19]. Thus, it is essential to understand the cardiovascular implant surface interaction with blood and its components and develop surfaces that can prevent protein adsorption and thrombus formation.

Several approaches have been investigated to develop the surfaces of cardiovascular implants with improved blood compatibility. Studies have shown that biocompatibility can depend on the implant surface topography, chemistry, crystallinity, and charge [20,21,22,23,24]. Surface passivation techniques such as mechanical treatment, plasma treatment, anodization, and thermochemical treatments modify the surface micro/nanoscale topography and chemistry [24,25,26]. These surfaces are normally hydrophilic and have been shown to influence hemocompatibility. While hydrophilic surface-induced interactions have proven to be highly advantageous for other implants, surface interaction for blood-contacting implants could also pose a great challenge due to thrombus formation [27,28]. Biopolymers, such as chitosan, heparin, and few zwitterionic polymers, are used to prevent protein adsorption and platelet adhesions, but they have not been found in the clinical market [29,30]. Further, recent studies have explored superhydrophobic surfaces for cardiovascular implants. Superhydrophobicity is only achieved with the combination of surface roughness/topography (e.g., micro, nano, or micro–nano topography) and surface chemistry (e.g., aminopropyltriethoxysilane, 3-acryloxypropyltrimethoxysilane + bis-1,2-(triethoxysilyl) ethane, heptadecafluoro-1,1,2,2-tetrahydrodecyl trichlorosilane). These surfaces have shown significant reductions in platelet and WBC adhesion [31,32,33,34,35]. However, nanotubes and nanoflowers developed on a titanium surface are shown to be instable and can easily delaminate. Hence, there is a need to develop a stable surface morphology that can lead to a superhydrophobic surface. Researchers have developed a superhydrophobic titanium surface using laser–hydrothermal treatment and PDMS/silane coating for anti-icing [36,37]. However, the hemocompatibility of these surfaces are not evaluated and the process on the micro–nano surface is complicated compared to the method reported in this manuscript.

In this study, a novel micro–nano surface topography was developed on a titanium surface using thermochemical technique with sulfuric acid. The surface was further modified with fluoro-silane to reduce the surface energy and make the surface superhemophobic. The surfaces were characterized for morphology using a scanning electron microscope (SEM); wettability with water, platelet rich plasma, and whole blood using a goniometer; and crystallinity using X-ray diffraction (XRD). The hemocompatibility of the surfaces was characterized by evaluating the fibrinogen adsorption, blood cell adhesion, platelet activation, hemolysis, thrombin generation, complement activation, and whole blood clotting. The results implied that the superhydrophobic micro–nano surface prevented platelet and WBC adhesion significantly (>90%) and prevented thrombus formation. Thus, this novel surface modification can be a potential cardiovascular implant surface with enhanced hemocompatibility.

## 2. Materials and Methods

### 2.1. Fabrication of Micro–Nanoporous Surfaces

The micro–nanoporous surfaces on Grade 2 titanium foils were fabricated using a thermochemical process and were further modified to be superhydrophobic with silane, as described in our previous work [38]. In brief, polished and cleaned titanium foils were thermochemically treated with 0.5 M sulfuric acid in a polytetrafluoroethylene container inside an 80 °C hot air oven for 8 h. After the treatment, the surfaces were ultrasonically cleaned and annealed at 300 °C for 1 h. The modified surfaces were washed with Milli-Q water, air dried, and kept in a sealed Petri dish inside a desiccator until further use. 

The micro–nanoporous surface was made superhydrophobic by grating with low-energy silane. Prior to grafting, the micro–nanoporous surfaces were plasma etched at 200 V in 10 cm^3^/min in an oxygen gas chamber for 5 min. Subsequently, the surfaces were modified by grafting with 150 μL of heptadecafluoro-1,1,2,2-tetrahydrodecyl trichlorosilane (silane) inside a closed chamber at 120 °C for 1 h.

The following notations are used for different surfaces in this manuscript: unmodified titanium surfaces: Ti; micro–nanoporous titanium surfaces: nTi; superhydrophobic micro–nanoporous surfaces: nTi-S. 

### 2.2. Surface Characterization

The topography of the control and modified surfaces was visualized using a JEOL 6500 scanning electron microscopy (SEM). The apparent contact angle (θ*) of the different surfaces with Milli-Q water, platelet-rich plasma (PRP), and human blood was calculated using the images taken with a Ramé–Hart goniometer. The presence of crystal structures on the surfaces was determined using a Bruker D8 discover DaVinci Powder X-ray diffraction (XRD) machine equipped with Cu Kα radiation. XRD scans were collected at θ = 1.5°. 

### 2.3. Surface Preparation for Hemocompatibility Studies 

The surfaces were cleaned in 24-well plates with Milli-Q water and phosphate-buffered saline (PBS), each for 5 min. They were further sterilized by exposure to ultraviolet light present in a biosafety cabinet for 15 min.

### 2.4. Isolation of Platelet-Rich Plasma (PRP) from Human Blood

Whole blood from healthy individuals who had refrained from using any medication that may affect blood clotting in past 7 days was collected in a blood collection tube containing ethylenediaminetetraacetic acid (EDTA). The procedure to collect the blood was performed as per the standard protocol authorized by the Institutional Review Board of Colorado State University and National Institutes of Health’s “Guiding Principles for Ethical Research”. Prior to the blood draw, consent was obtained from the human participants. The first tube of blood was discarded to avoid the use of locally activated platelets due to needle insertion. The collected blood was centrifuged at 150× *g* for 15 min. The upper layer and buffy coat (platelet-rich plasma) from the tubes were pooled prior to using with different surfaces after resting for another 15 min.

### 2.5. Fibrinogen Binding on Different Surfaces from PRP

The fibrinogen binding on different surfaces from the pooled PRP was measured using a commercially available enzyme-linked immunoassay (ELISA). The sterilized surfaces were incubated with PRP for 2 h on a flat shaker at 100 rpm, 37 °C, and 5% CO_2_. The PRP incubated with the surfaces was diluted 1/10,000-fold and the manufacturer-provided protocol was followed to determine the fibrinogen binding on the different surfaces. The absorbance of the final solution was measured using a microplate reader at a 450 nm wavelength. The results presented were calculated after subtracting the positive control (total fibrinogen present after incubation with empty well).

### 2.6. Cell Adhesion on Different Surfaces

The cell adhesion from PRP on the different surfaces was visualized using fluorescence microscopy. The sterilized surfaces were incubated with PRP for 2 h on a flat shaker at 100 rpm, 37 °C, and 5% CO_2_. After incubation, the surfaces were isolated and rinsed thrice with PBS. The surfaces were then incubated with 5% calcein-AM stain for 20 min. The stain solution was aspirated and the surfaces were rinsed thrice with PBS. The surfaces were imaged using a Zeiss fluorescence microscope. All images were processed, and the area covered by the live cells was measured using ImageJ.

### 2.7. Identification of Platelets and WBCs on Different Surfaces

The identification of platelets and WBCs on the different surfaces was achieved using fluorescence microscopy. The sterilized surfaces were incubated with PRP for 2 h on a flat shaker at 100 rpm, 37 °C, and 5% CO_2_. After incubation, the surfaces were isolated and rinsed thrice with PBS. The rinsed surfaces were fixed using formaldehyde (3.7%) and were rinsed thrice with PBS. The cells were further permeabilized using Triton (1%) and were rinsed four times with PBS. The surfaces were then incubated with 0.05% rhodamine−phalloidin (actin)stain solution and incubated for 25 min. Later, 4′,6-diamidino-2- phenylindole (DAPI) stain solution (3%) was added and incubated for 5 min. The stain solution was aspirated, and the surfaces were rinsed thrice with PBS. The surfaces were imaged using a Zeiss fluorescence microscope. All images were further processed and the number of cells determined using ImageJ.

### 2.8. Platelet Activation on Different Surfaces

The platelet activation and platelet/WBC complex formation on the different surfaces was visualized using SEM. The sterilized surfaces were incubated with PRP for 2 h on a flat shaker at 100 rpm, 37 °C, and 5% CO_2_. After incubation, the PRP solution was aspirated and the surfaces were rinsed thrice with PBS. The surfaces were then fixed using a fixative solution containing glutaraldehyde (6%), 0.1 M sodium cacodylate, and 0.1 M sucrose for 45 min. Later, the surfaces were then incubated in a buffer solution containing 0.1 M sodium cacodylate and 0.1 M sucrose for 10 min. This was followed by the dehydration of the surfaces by incubation in 35, 50, 70, and 100% ethanol for 10 min each. The surfaces were dried and imaged using SEM. 

### 2.9. Hemolysis of Erythrocytes on Different Surfaces

The hemolytic activity on different surfaces was measured using a commercially available hemolysis assay kit (HaemoScan, Groningen, Netherlands), which is in accordance with the international standard ISO 10993/Part 4 and ASTM F756-08 standards [39] to evaluate the hemocompatibility of biomaterials. The sterilized surfaces and control surfaces (Buna-S and silicon elastomer, provided with the assay) were incubated with the manufacturer-provided erythrocyte suspension for 24 h on a flat shaker at 100 rpm, 37 °C, and 5% CO_2_. The protocol provided by the manufacturer was followed to determine the hemolytic activity on the different surfaces. The absorbance of the resulting solution was measured at different wavelengths (415/450/380 nm) using a microplate reader.

### 2.10. Thrombin Generation on Different Surfaces

The thrombin generation on the different surfaces was measured using a commercially available thrombin generation assay (HaemoScan, Groningen, Netherlands), which is in accordance with the international standard ISO 10993/Part 4 to evaluate the biocompatibility of biomaterials. The sterilized surfaces and manufacturer-provided control surfaces (low-density polyethylene and medical steel, provided with the assay) were incubated with the manufacturer-provided plasma for 15 min at 37 °C. The manufacturer’s protocol was followed to determine the maximum thrombin generation over a time interval and the average thrombin generation over a period of 4 min from the solution incubated with the different surfaces. The absorbance of the resulting solution was measured at different wavelengths (405/540 nm) using a microplate reader.

### 2.11. Complement Convertase on Different Surfaces

The complement activation on the different surfaces was measured using a commercially available complement convertase kit (HaemoScan, Groningen, Netherlands), which is in accordance with the international standard ISO 10993/Part 4 to evaluate the biocompatibility of biomaterials. The sterilized surfaces and manufacturer-provided control surfaces (medical steel, polydimethylsiloxane, and low-density polyethylene, provided with the assay) were incubated with the manufacturer-provided plasma for 24 h at 37 °C and 5% CO_2_. The manufacturer’s protocol was followed to determine the complement generated due to the interaction with different surfaces. The absorbance of the resulting solution was measured at a 405 nm wavelength using a microplate reader.

### 2.12. Whole Blood Clotting on Different Surfaces

The whole blood clotting on the different surfaces was assessed by quantifying the free hemoglobin in unclotted blood after the exposure of the whole human blood to the surfaces. The blood was collected in a tube without any anticoagulant coating and the study was performed immediately after the blood draw. In total, 5 µL of whole blood was pipetted on top of the different surfaces, and the blood was allowed to clot for up to 45 min. After every 15 min, the surfaces were evaluated for the presence of free hemoglobin. Milli-Q water was added to the surfaces and gently shaken for 30 s to lyse the RBCs that were not trapped in the clot on the surface. The absorbance of free hemoglobin released by the lysed RBCs was measured using a microplate reader at 540 nm. 

### 2.13. Statistical Analysis 

Surface characterization was performed using three different samples of each surface. The surface morphology, using SEM and wettability using a goniometer, were taken at three different locations on each sample (n_min_ = 9). Different hemocompatibility studies were repeated at least three times with blood from at least two heathy individuals (n_min_ = 6). The result presented is from a single study with blood drawn from single individual. This is because there is a significant difference in the platelet count from each individual and it is not appropriate to compare the absolute value. However, similar trends were observed for blood from different donors/assay kits for the results presented, indicating the reproducibility of the data. The quantitative data were processed using R software and the p-value was determined using a two-way analysis of variance (ANOVA) test. For a *p*-value < 0.05, the dataset was considered significant. The error bar represents the standard deviation.

## 3. Results and Discussion

Even though extensive research has been carried out for enhancing implants’ surface interaction with blood and its components, to date, a truly hemocompatible implant surface that can prevent blood clotting has not been developed. Studies have shown that the implant surface’s interaction with blood stimulates protein adsorption (fibrinogen), platelet adhesion, platelet activation, and inflammatory reactions. These improper reactions affect implant functionality and life. Hence, in this study, a novel superhydrophobic surface was developed and its hemocompatibility was characterized by quantifying the fibrinogen protein adsorption, hemolysis, thrombin generation, complement activation, and platelet adhesion/activation. Previous studies have demonstrated that these surfaces have good corrosion resistance and antibacterial properties [38].

**Figure 1:**
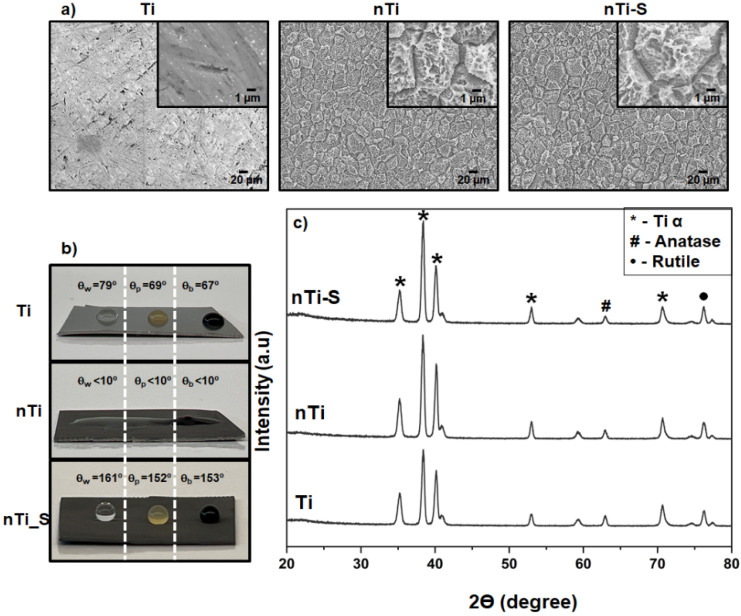
(**a**) Representative SEM images of different surfaces. Images were taken at 500×, and image inserts depict 5000× magnification. (**b**) Apparent contact angle measurements using Milli-Q water, PRP, and blood on different surfaces. (**c**) XRD intensity peaks of different surfaces (n_min_ = 9).

The topography of the different surfaces was imaged using SEM. Surface topography is an important property that determines the biological responses to a foreign material. The thermochemical treatment alters the surface properties such as the topography, chemistry, and wettability without altering the bulk properties of the titanium substrate [40]. The results (Figure 1a) indicated that the Ti surfaces were smooth and did not have any unique surface topography. The nTi surfaces had a microscale topography formed because of quicker etching near the grain boundaries. Faster etching is due to the disordered atomic arrangement at the grain boundaries. Further, etching on the grain surfaces led to the formation of nano-pits. Thus, the results indicate that the thermochemical treatment led to a micro–nano topography. Further, after silane modification, the nTi-S surfaces did not show any significant difference in the surface topography when compared to the nTi surfaces. The presence of silane after deposition was confirmed with the XPS results in the author’s previous publication [38].

The wettability of the different surfaces was quantified by measuring the apparent contact angle with Milli-Q water (θw), PRP (θp), and whole blood (θb) using a goniometer. Studies have shown that blood is mostly made up of water. Wettability can be broadly classified into three categories: superhemo/hydrophobic when θw & θb > 150°, hemo/hydrophobic if θw & θb > 90°, and hemo/hydrophilic if θw & θb < 90°. Liquid interaction with a textured surface can adapt two configurations to reduce the total liquid–solid free energy: the Wenzel and the Cassie–Baxter state. At the Wenzel state, the fluid permeates into the surface topography and increases the liquid–solid interfacial area. This reduces the apparent contact angle compared to the surface without surface features. Contrarily, in the meta-stable Cassie–Baxter state, the liquid does not interact with the surface topography, creating air pockets between the liquid and surface topography. This leads to a high apparent contact angle. Studies have shown that the Cassie–Baxter state can be achieved with a combination of low-energy surface chemistry and appropriate surface topography [27]. Previous studies have shown that a silane coating on a plain polished titanium surface is not superhydrophobic. The results (Figure 1b) indicated that, as expected, the Ti surface is hydro/hemophilic; this is because of the absence of any unique topography on the surface and the high apparent contact angle compared to the Milli-Q water, PRP, and whole blood [6,33]. The nTi surface is super-hemo/hydrophilic because of a combination of micro–nano surface topography and higher titanium oxide, as confirmed with the XPS results in a previous publication [38]. Studies have shown that the surface oxide layer attracts water molecules. However, the nTi-S surface is super-hemo/hydrophobic surface in the Cassie–Baxter state, as confirmed by advancing contact angle studies from wettability studies in previous research [38]. This due to the presence of low-energy silane on the surface and the micro–nano topography. The apparent contact angle with blood was lower compared to PRP because blood is a denser liquid than PRP. 

The phase analysis of the different surfaces was assessed using XRD. Studies have shown that surface crystallinity plays a major role in wettability and cellular interaction [41,42]. The rutile and anatase phase of TiO_2_ was present on all surfaces and these phases have been shown to be more cytocompatible. The results (Figure 1c) indicated that the surfaces have the following metallic alpha-phase titanium intensity peaks at 32° (002), 35° (100), 40° (101), and 53° (102). The TiO_2_ anatase-phase intensity peaks at 62° (204), and the TiO_2_ rutile-phase intensity peaks at 27° (110) and 76° (110). The nTi and nTi-S had a higher presence of metallic alpha-phase titanium on the surface because the treatment removed the impurities and the oxide layer on the Ti surface. The surface modification of nTi-S did not alter the crystal structure when compared to the nTi surface. 

**Figure 2:**
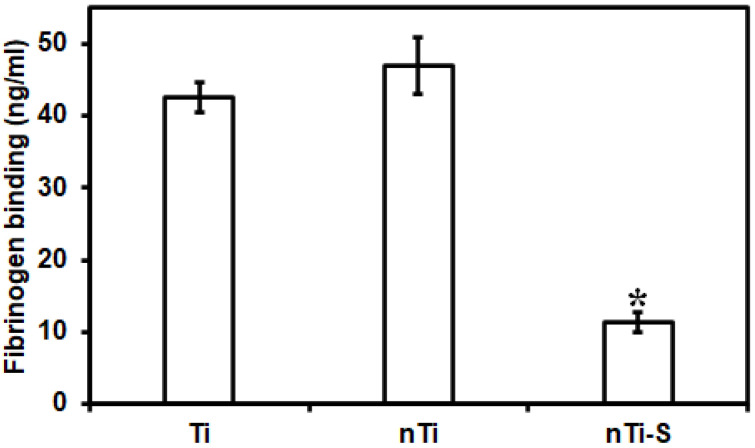
Fibrinogen adsorption from PRP on different surfaces measured using a microplate reader. (* *p* < 0.05; n_min_ = 9.)

The fibrinogen adsorption from PRP on the different surfaces was quantified using a commercially available enzyme-linked immunoassay (ELISA) for human fibrinogen. The most abundant proteins present in human blood are albumin and fibrinogen. Albumin is a passivating protein; it does not actively promote blood clotting. However, fibrinogen is a key protein in the development of surface-induced thrombosis through binding the integrin receptor αIIbβ3 (GPIIb/IIIa) of platelets. This leads to platelet immobilization, activation, and aggregation, and this is also a precursor for fibrin formation. Fibrin is a leading structural component in the blood clotting coagulation cascade. Various studies have demonstrated that nano, micro, or micro–nano surface topography influences protein adsorption and alters protein conformation and spatial distribution on the surface [17,43]. The sizes of the proteins are in the nanometer range and studies have shown that the surface topography and its feature size can influence protein adsorption and nucleation within the topography [44]. PRP mostly contains blood proteins (fibrinogen), platelets, and white blood cells. The surface-exposed PRP was analyzed to evaluate the protein content in the solution, and the amount of protein adsorbed on the surface was calculated by subtracting the protein content in the positive control. The results (Figure 2) indicate no significant difference in fibrinogen adsorption on the nTi and Ti surfaces. On the contrary, the nTi-S surfaces had significantly lower fibrinogen adsorption compared to the nTi and Ti surface. This is because the lower surface energy nTi-S surface prevents the interaction with liquid, reducing the protein adsorption. In addition, studies have shown that fibrinogen adhered to hydrophobic surfaces has a specific conformation that prevents fibrinogen fiber formation [45].

**Figure 3:**
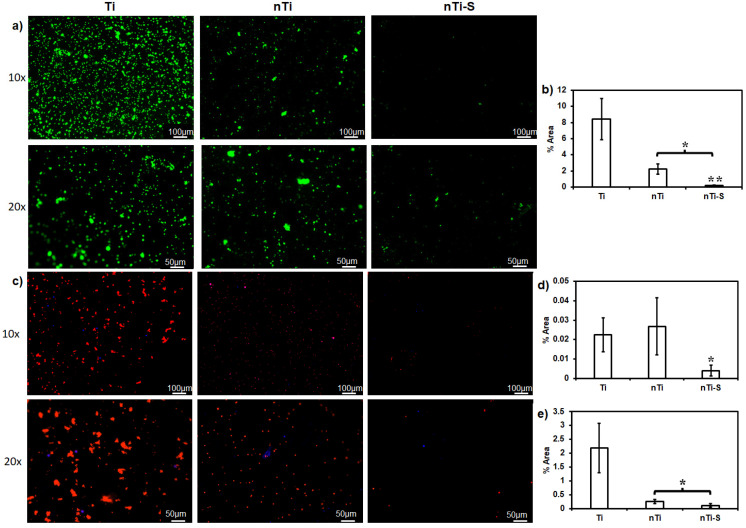
(**a**) Fluorescence images of live cells (platelets and WBCs) adhered to different surfaces. (**b**) Percentage area covered by adhered platelets and WBCs on different surfaces. (**c**) Fluorescence images of adhered platelets (red) and WBCs (purple) on different surfaces. (**d**) Percentage of the areas covered by adhered platelets (red) on different surfaces. (**e**) Percentage area covered by adhered WBCs (blue) on different surfaces. (* and ** *p* < 0.05; n_min_ = 9.)

The live cell adhesion (platelets and WBCs) on the different surfaces was assessed after incubation with PRP. The surfaces with adhered live cells were stained with calcein-AM. Platelet and WBC adhesion is the immediate step in the coagulation cascade after fibrinogen adsorption on the surface [46,47]. Activated platelets further promote platelet adhesion and activation and simultaneously interact with WBCs (Figure 3a), leading to platelet/WBC complex formation. The results (Figure 3b) indicated that the Ti surface adhered to significantly more cells compared to the nTi and nTi-S surfaces. The nTi surface showed lower cell adhesion compared to the Ti surface because it has a significantly lower surface area of contact due to the presence of nano-pits, thus preventing platelets/WBCs from adhering to the surface. However, the Ti surface is a simple planar surface, which alleviates platelet/WBC adhesion. The nTi-S surface had the lowest platelet/WBC adhesion. This is because the superhemophobic nature of the surface prevents any interaction of the liquid with the surface.

The identification of the platelets and WBCs on the different surfaces was achieved after incubation with PRP by staining with rhodamine phalloidin and DAPI. DAPI purple stains the nuclei of the cells, whereas rhodamine phalloidin red stains the cytoskeleton of the cells. Since platelets do not have nuclei, they are stained red. Platelets play a crucial role in the extrinsic pathway of the coagulation cascade, while WBCs play a crucial role in the intrinsic pathway by secreting anticoagulant molecules and indirectly activating platelets. WBC and platelet complex formation can accelerate thrombus formation [48]. Rhodamine phalloidin stains positive for the cytoskeleton of both platelets and WBCs. DAPI stains positive for the nucleus of WBCs and negative for platelets, as they are anuclear (Figure 3c). The WBC adhesion results (Figure 3d) showed that both Ti and nTi had significantly higher WBC adhesion compared to nTi-S. The platelet adhesion results (Figure 3e) indicated that the Ti surface had significantly higher platelet adhesion compared to the nTi and nTi-S surfaces. nTi showed lower platelet adhesion compared to the Ti surface because the micro–nano surface topography had localized platelet adhesion and prevented platelet aggregation. The nTi-S surface had the lowest platelet/WBC adhesion. This is because the superhemophobic surface significantly prevents blood component interactions. 

**Figure 4:**
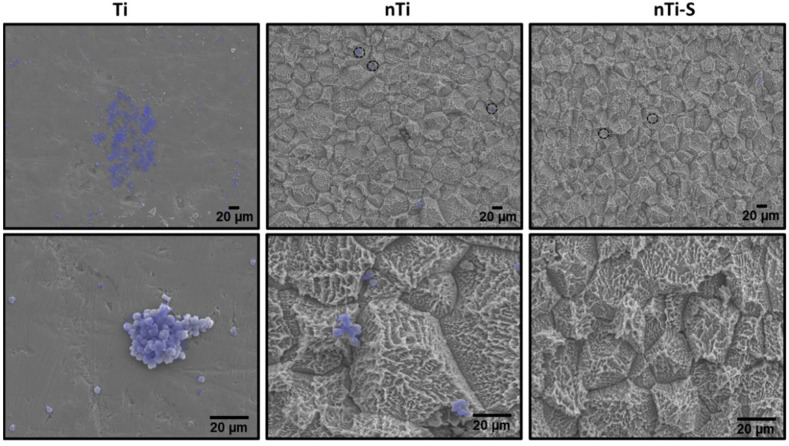
Representative SEM images of adhered platelets and WBCs (purple—Photoshop used for better visibility) on different surfaces. Images were taken at 500× and 2000× magnification. (n_min_ = 9.)

The platelet and WBC adhesion, activation, and complex aggregate formation on the different surfaces were visualized after incubation with PRP using SEM. When activated, platelets go through a morphology change and form dendrites and initiate aggregation. The activated platelet indirectly supports WBC localization during thrombosis. Simultaneously, the platelet/WBC complex formation promotes inflammation reactions. Activated platelets alter their morphology and develop dendrites on the peripheral region [49]. The results (Figure 4) indicated that the Ti surface exhibits significantly promoted platelet adhesion and activation (dendrite formation) and the planar surface enables easy platelet aggregation and platelet/WBC formation (highlighted in purple). The nTi surface shows lower platelet adhesion and aggregate formation compared to the Ti surface. THe nTi surface does have platelet adhesion; however, the surface morphology prevents well-spread platelet aggregation and platelet activation (dendrite formation). The nTi-S surface had the least platelet adhesion, and neither platelet aggregation nor platelet activation is observed on the surface.

**Figure 5:**
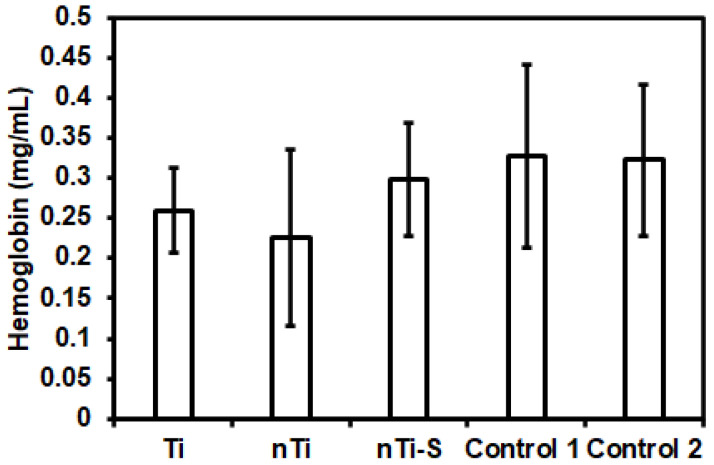
Hemoglobin release from erythrocytes solution incubated with different surfaces was measured using a microplate reader. (n_min_ = 9.)

The hemolytic activity of the different surfaces was assessed after incubation with erythrocytes using a commercially available hemolysis assay. Studies have shown that erythrocyte lysis can be induced due to contact with the implant surface due to its surface chemistry, surface charge, and topography [6]. The erythrocyte lysis induced by surface interactions leads to the release of hemoglobin [50,51]. Therefore, the presence of hemoglobin is a marker of hemolysis. The results (Figure 5) indicated that the hemoglobin release from the erythrocytes incubated with different surfaces was not significantly different from the two FDA-approved Buna N (control 1) and silicon elastomer (control 2). Hence, neither the micro–nano surface topography nor the silane chemistry induces hemolysis.

**Figure 6:**
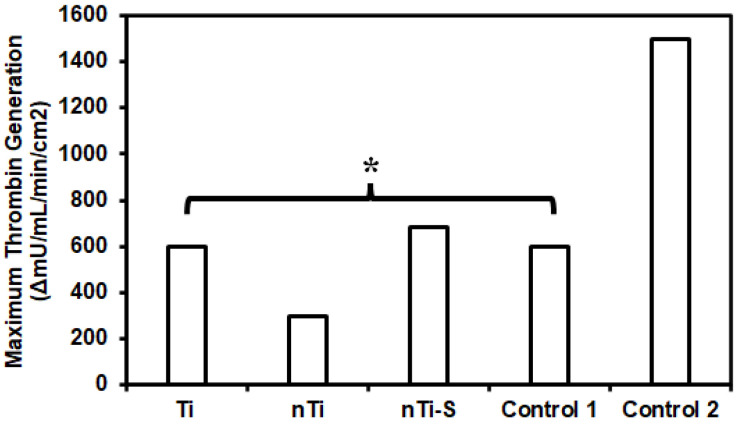
Highest thrombin generation velocity of plasma incubated with different surfaces between two points. (* *p* < 0.05, n_min_ = 9).

The thrombin formation of different surfaces was assessed using a commercially available thrombin generation assay. Studies have shown that thrombin plays a vital role in converting fibrinogen to fibrin, which is an integral step in clot formation. Thrombin is formed from prothrombin due to the coagulation cascade formation from the activation of both the intrinsic and extrinsic pathway [4]. Thrombin generation also activate platelets and inflammatory cell chemotaxis. Thrombin has a short half-life, making it complicated to quantify its activity [52]. The surface-exposed manufacturer-provided plasma was activated, and thrombin generation was measured at different time points. The assay is developed to measure the thrombin generation velocity at different time points and the highest velocity is reported. Standard deviation is applicable for this assay. The results (Figure 6) indicated that the rate at which thrombin was generated was significantly higher with medical steel compared to low-density polyethylene, titanium, and other modified surfaces over a period of 4 min. The nTi surface had the lowest thrombin generation velocity compared to Ti, nTi-S, low-density polyethylene (control 1), and medical steel (control 2) surfaces. This may be because the nTi surface is hydrophilic and there is constant thrombin generation.

**Figure 7:**
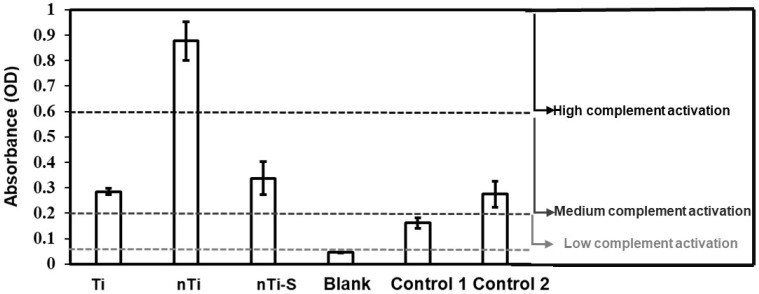
Complement activation of plasma incubated with different surfaces was measured as activation of complement convertase C5a. The lines indicate the regions of inactive/low (≤0.2), medium (>0.2 and ≤0.6), and high (>0.6) reactivity. (n_min_ = 9.)

The complement convertase activation of different surfaces was assessed using a commercially available complement convertase assay. Thrombus formation is due to the initiation of the coagulation cascade, and this cascade is divided into two categories: the intrinsic and extrinsic pathway. The intrinsic pathway (contact activation) is because of the interaction between the adsorbed proteins and the implant surface. Complement activation is a part of the intrinsic inflammatory response and simultaneously influences blood clotting. Complement activation alleviates WBC adhesion and activation on the implant surface [53,54]. The different surfaces were incubated with manufacturer-provided plasma and the adhered complement factors on the surface were measured. The results (Figure 7) indicate that the nTi surface had significantly higher complement activation compared to the other modified, low-density polyethylene (control 1), and medical steel (control 2) surfaces. However, there was no significant difference between the medical steel, Ti, and nTi-S surfaces and the values were in the lower spectrum of medium complement activation.

**Figure 8:**
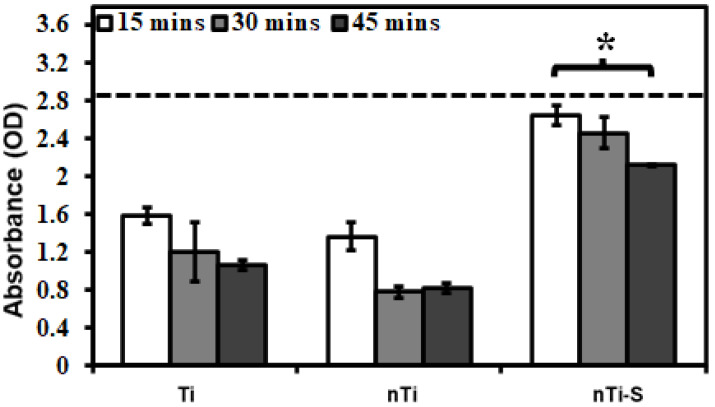
Whole blood clotting on different surfaces for up to 45 min. The dotted line represents the absorbance of free hemoglobin in un-clotted blood. (* *p* < 0.05; n_min_ = 9.)

The blood clotting kinetics on the different surface was assessed after incubation with whole blood by quantifying the free hemoglobin in the blood interacting with the surface. Previous studies evaluated the influence of proteins, enzymes, and cells individually with the modified surfaces. However, when an implant is placed inside the human body, whole blood comes into contact with it. Hence, the whole blood clotting kinetics was studied. After 15 min, 30 min, and 45 min of incubation, the different surfaces were immersed in Milli-Q water to measure the hemoglobin on the live RBCs. RBCs, in the presence of Milli-Q water, rupture because of osmosis releasing the hemoglobin. Thus, a higher concentration of hemoglobin in Milli-Q water indirectly indicated less blood clotting at the surface. The results (Figure 8) indicated that after 45 min, the nTi-S surface had significantly higher hemoglobin compared to the Ti and nTi surfaces, thus evidently preventing blood clotting significantly. Similar trends were observed over the entire duration of the study. Over time, there was a steady decrease in free hemoglobin; a part of this is due to the exposure of blood to the atmosphere.

## 4. Conclusions

Blood-contacting implants are highly susceptible to thrombosis and bacterial infection as the planar surface properties alleviate improper interactions. In the quest of developing hemocompatible surfaces, a thermochemical method was explored for modifying implant surfaces; this technique is very elementary and is easily scalable. In this present study, titanium substrates were thermochemically treated with sulfuric acid under a controlled atmosphere. The treatment etched the surface at the micron and nano scale.

(a) The treatment led to a novel micro–nano surface topography, and further coating with silane made the surface superhemophobic.
(b) Hemolysis studies show that there was no significant hemolysis due to the surface modifications compared to the reference materials. The fibrinogen adhesion from PRP shows that the superhemophobic surface adhered significantly lower fibrinogen compared to the other surfaces. Studies have shown that reduced fibrinogen adhesion prevents platelet adhesion and activation.
(c) The Ti and modified surfaces showed significantly lower thrombus generation kinetics compared to medical grade steel. The modified superhemophobic surface did not activate higher complement, thus preventing any inflammatory reactions. However, the micro–nano surface showed higher complement activation.
(d) The superhemophobic surface significantly prevented platelet/WBC adhesion compared to other surfaces. However, the micro–nano surface had similar WBC adhesion and lower platelet adhesion compared to Ti. The planar Ti surface had significantly higher platelet adhesion, activation, and platelet/WBC complex formation. However, the micro–nano surface showed localized platelet adhesion and aggregate formation, but prevented platelet/WBC complex formation due to its surface topography. On the contrary, the superhemophobic surface proved to be the most hemocompatible surface as it significantly prevented platelet and WBC adhesion.
(e) The whole blood clotting kinetics showed that the superhemophobic surface had significantly lower whole blood clotting after 45 min of incubation compared to the other surfaces. Thus, the micro–nano surface coated with silane is a promising candidate for blood-contacting implant application.

Further studies are directed towards analyzing the mechanisms causing the improved hemocompatibility of micro/nano surface features and stability by investigating the influence of superhemophobic surface dynamics under in vitro and in vivo conditions.

## 5. Patents

This work is part of a patent application.

## Data Availability

The data presented in this study are available on request from the corresponding author. The data are not publicly available due to ethical reasons.
